# A chiral photochromic Schiff base: (*R*)-4-meth­oxy-2-[(1-phenyl­ethyl)imino­meth­yl]phenol

**DOI:** 10.1107/S1600536809035557

**Published:** 2009-09-09

**Authors:** Yukie Miura, Yoshikazu Aritake, Takashiro Akitsu

**Affiliations:** a Department of Chemistry, Faculty of Science, Tokyo University of Science, 1-3 Kagurazaka, Shinjuku-ku, Tokyo 162-8601, Japan

## Abstract

The title chiral photochromic Schiff base compound, C_16_H_17_NO_2_, was synthesized from (*R*)-1-phenyl­ethyl­amine and 5-methoxy­salicylaldehyde. The mol­ecule of the title compound exists in the phenol–imine tautomeric form. The dihedral angle between the two aromatic rings is 62.61 (11)°. An intra­molecular O—H⋯N hydrogen bond with an O⋯N distance of 2.589 (2) Å is observed. The crystal packing is stabilized by C—H⋯π inter­actions involving the aromatic ring.

## Related literature

For chiral metal complexes and their hybrid materials, see: Akitsu (2007 ▶); Akitsu & Einaga (2004 ▶, 2005*a* ▶,*b* ▶, 2006*a* ▶); Akitsu *et al.* (2009 ▶); Yamada (1999 ▶). For structral comparison of the 1-phenylethylamine moiety, see: Antonov *et al.* (1995 ▶); Liu *et al.* (1997 ▶). For related Schiff base ligands and their functions, see: Akitsu *et al.* (2004 ▶); Akitsu & Einaga (2006*b* ▶); Hadjoudis *et al.* (1987 ▶, 2004 ▶); Santoni & Rehder (2004 ▶); Sliwa *et al.* (2005 ▶). 
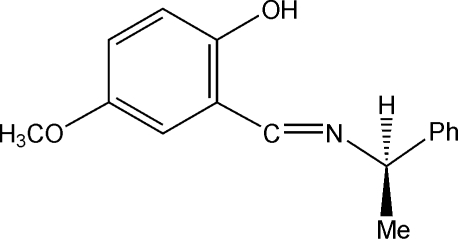


## Experimental

### 

#### Crystal data

C_16_H_17_NO_2_
*M* *_r_* = 255.31
Monoclinic, 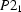

*a* = 8.270 (4) Å
*b* = 5.886 (3) Å
*c* = 13.920 (7) Å
β = 93.254 (7)°
*V* = 676.4 (6) Å^3^
*Z* = 2
Mo *K*α radiation
μ = 0.08 mm^−1^
*T* = 100 K
0.21 × 0.19 × 0.07 mm

#### Data collection

Bruker SMART CCD area-detector diffractometer
Absorption correction: multi-scan (*SADABS*; Bruker, 1998 ▶) *T*_min_ = 0.983, *T*_max_ = 0.994
3805 measured reflections
1677 independent reflections
1454 reflections with *I* > 2σ(*I*)
*R*_int_ = 0.074

#### Refinement

*R*[*F* ^2^ > 2σ(*F* ^2^)] = 0.042
*wR*(*F* ^2^) = 0.088
*S* = 0.99
1677 reflections
240 parameters
1 restraint
All H-atom parameters refined
Δρ_max_ = 0.31 e Å^−3^
Δρ_min_ = −0.18 e Å^−3^

### 

Data collection: *SMART* (Bruker, 1998 ▶); cell refinement: *SAINT* (Bruker, 1998 ▶); data reduction: *SAINT*; program(s) used to solve structure: *SHELXS97* (Sheldrick, 2008 ▶); program(s) used to refine structure: *SHELXL97* (Sheldrick, 2008 ▶); molecular graphics: *SHELXTL* (Sheldrick, 2008 ▶); software used to prepare material for publication: *SHELXL97*.

## Supplementary Material

Crystal structure: contains datablocks global, I. DOI: 10.1107/S1600536809035557/ci2897sup1.cif

Structure factors: contains datablocks I. DOI: 10.1107/S1600536809035557/ci2897Isup2.hkl

Additional supplementary materials:  crystallographic information; 3D view; checkCIF report

**Fig. 1.**
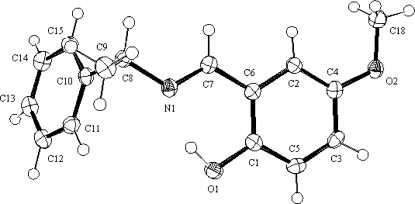
The molecular structure of (I), showing the atom-labelling scheme. Displacement ellipsoids are drawn at the 50% probability level.

Enhanced figure: interactive version of Fig. 1

## Figures and Tables

**Table 1:** Hydrogen-bond geometry (Å, °)

*D*—H⋯*A*	*D*—H	H⋯*A*	*D*⋯*A*	*D*—H⋯*A*
O1—H1⋯N1	0.97 (3)	1.72 (5)	2.589 (2)	151 (3)
C12—H12⋯*Cg*1^i^	1.03 (4)	2.72 (3)	3.536 (3)	137 (3)
C16—H16*C*⋯*Cg*1^ii^	0.98 (4)	2.71 (3)	3.563 (3)	149 (3)

Symmetry codes: (i) 
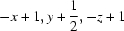
; (ii) 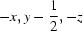. *Cg* 1 is the centroid of the C10–C15 ring.

## supplementary crystallographic information

### Comment

Because of structural flexibility and their application for switching materials and so on, Schiff base compounds are one of the most extensively used ligands in the field of coordination chemistry (Yamada, 1999). Especially, aiming at multifunctional chiral materials, we have investigated Schiff base Cu^II^, Ni^II^, or Zn^II^ complexes in view of thermally induced structural phase transition in the solid state (Akitsu & Einaga, 2004), structural change by occlusion of solvents (Akitsu & Einaga, 2005*a*), chiral conformational change in a solution induced by a photochromic solute (Akitsu & Einaga, 2005*b*,2006*a*; Akitsu, 2007), and novel induced CD to achiral metallodendrimers (Akitsu *et al.*, 2009). On the other hand, free Schiff base ligands (Akitsu *et al.*, 2004, Akitsu & Einaga, 2006*b*) have been also studied as multifunctional components, for example photochromic and thermochromic or fluorescence materials (Hadjoudis *et al.*, 2004) and nonlinear optical materials (Sliwa *et al.*, 2005) and so on. In order to clarify the role of electron-donating methoxy group, as free ligands for tautomerism and photochromism (Hadjoudis *et al.*, 1987), crystal structure of the title compound, (I), has been determined.

Crystal structure of (I) is similar to those of the analogous derivatives (Santoni & Rehder, 2004; Akitsu & Einaga, 2006*b*). Molecule of (I) (Fig. 1) adopts an E configuration with respect to the imine C═N double bond with a C6—C7—N1—C8 torsion angle of -179.40 (18)°. Thus, the π-conjugate system around the imine group is essentially planar. The C1—O1 bond distance of 1.361 (3) Å suggests that it is in the phenol-imine tautomer. The contraction of the C7═N1 bond [1.283 (3) Å] is also in agreement with the phenol-imine tautomer. As for the methoxy group, the O2—C4 and O2—C16 bond distaces are 1.374 (3) and 1.422 (3) Å, respectively, and the C4—O2—C16 bond angle is 116.9 (2)°. Beside them, geometric parameters reported here agree with corresponding values reported for analogous Schiff base compounds containing the 1-phenylethylamine group (Antonov *et al.*, 1995; Liu *et al.*, 1997). The planarity of (I) is stabilized by an intramolecular O—H···N hydrogen bond (Table 1). However, there is no intermolecular hydrogen bonds associated with the methoxy group. The crystal packing is stabilized by C—H···π interactions involving the C10-C15 ring.

### Experimental

Treatment of equimolar *R*-1-phenylethylamine and 5-methoxysalicylaldehyde in methanol at 298 K overnight gave rise to a yellow-green compound (I). Prismatic crystals of (I) were grown from the resulting solution over a period of several days (yield 39.0%). Analysis found: C 73.98, H 6.49, N 5.37%; calculated for C_16_H_17_NO_2_: C 75.27, H 6.71, N, 5.49%. (precipitates containing non-stoichiometric cystalline water) m.p. 371 K. IR (Nujol, ν, cm^-1^): 1632 (imine band). UV-VIS (diffuse reflectance, nm): 255, 329, 470s h.

### Refinement

All H atoms were located in a difference map and refined freely [O-H = 0.98 (3) Å and C-H = 0.91 (3)-1.02 (3) Å]. Friedel pairs were merged.

### Crystal data

**Table:**

C_16_H_17_NO_2_	*F*(000) = 272
*M**_r_* = 255.31	*D*_x_ = 1.253 Mg m^−^^3^
Monoclinic, *P*2_1_	Mo *K*α radiation, λ = 0.71073 Å
Hall symbol: P 2yb	Cell parameters from 1657 reflections
*a* = 8.270 (4) Å	θ = 2.5–27.5°
*b* = 5.886 (3) Å	µ = 0.08 mm^−^^1^
*c* = 13.920 (7) Å	*T* = 100 K
β = 93.254 (7)°	Plate, yellow
*V* = 676.4 (6) Å^3^	0.21 × 0.19 × 0.07 mm
*Z* = 2	

### Data collection

**Table:**

Brruker SMART CCD area-detector diffractometer	1677 independent reflections
Radiation source: fine-focus sealed tube	1454 reflections with *I* > 2σ(*I*)
graphite	*R*_int_ = 0.074
Detector resolution: 8.333 pixels mm^-1^	θ_max_ = 27.5°, θ_min_ = 1.5°
φ and ω scans	*h* = −10→8
Absorption correction: multi-scan (*SADABS*; Bruker, 1998)	*k* = −7→7
*T*_min_ = 0.983, *T*_max_ = 0.994	*l* = −17→16
3805 measured reflections	

### Refinement

**Table:**

Refinement on *F*^2^	Primary atom site location: structure-invariant direct methods
Least-squares matrix: full	Secondary atom site location: difference Fourier map
*R*[*F*^2^ > 2σ(*F*^2^)] = 0.042	Hydrogen site location: inferred from neighbouring sites
*wR*(*F*^2^) = 0.088	All H-atom parameters refined
*S* = 0.99	*w* = 1/[σ^2^(*F*_o_^2^) + (0.038*P*)^2^] where *P* = (*F*_o_^2^ + 2*F*_c_^2^)/3
1677 reflections	(Δ/σ)_max_ = 0.001
240 parameters	Δρ_max_ = 0.31 e Å^−^^3^
1 restraint	Δρ_min_ = −0.18 e Å^−^^3^

### Special details

**Table:**

Experimental. Refinement of F^2^ against ALL reflections. The weighted *R*-factor *wR* and goodness of fit S are based on F^2^, conventional *R*-factors *R* are based on F, with F set to zero for negative F^2^. The threshold expression of F^2^ > 2sigma(F^2^) is used only for calculating *R*-factors(gt) *etc*. and is not relevant to the choice of reflections for refinement. *R*-factors based on F^2^ are statistically about twice as large as those based on F, and R– factors based on ALL data will be even larger.
Geometry. All e.s.d.'s (except the e.s.d. in the dihedral angle between two l.s. planes) are estimated using the full covariance matrix. The cell e.s.d.'s are taken into account individually in the estimation of e.s.d.'s in distances, angles and torsion angles; correlations between e.s.d.'s in cell parameters are only used when they are defined by crystal symmetry. An approximate (isotropic) treatment of cell e.s.d.'s is used for estimating e.s.d.'s involving l.s. planes.

### Fractional atomic coordinates and isotropic or equivalent isotropic displacement parameters (Å^2^)

**Table:**

	*x*	*y*	*z*	*U*_iso_*/*U*_eq_	
O1	−0.10235 (18)	0.3421 (3)	0.24677 (11)	0.0246 (4)	
O2	−0.34502 (18)	0.0560 (3)	−0.11019 (11)	0.0236 (4)	
N1	0.0495 (2)	−0.0409 (3)	0.26781 (13)	0.0209 (4)	
C1	−0.1599 (2)	0.2610 (4)	0.15989 (16)	0.0204 (5)	
C2	−0.2717 (3)	0.3907 (4)	0.10592 (17)	0.0235 (5)	
C3	−0.3316 (2)	0.3147 (4)	0.01743 (17)	0.0214 (5)	
C4	−0.2782 (2)	0.1095 (4)	−0.02039 (15)	0.0202 (5)	
C5	−0.1684 (3)	−0.0231 (4)	0.03255 (16)	0.0200 (5)	
C6	−0.1087 (2)	0.0500 (4)	0.12470 (15)	0.0190 (5)	
C7	0.0002 (2)	−0.0964 (4)	0.18197 (16)	0.0198 (5)	
C8	0.1607 (3)	−0.1916 (4)	0.32334 (16)	0.0204 (5)	
C9	0.0764 (3)	−0.2731 (5)	0.41192 (19)	0.0253 (6)	
C10	0.3138 (2)	−0.0565 (4)	0.34822 (15)	0.0200 (5)	
C15	0.4606 (3)	−0.1240 (5)	0.31292 (16)	0.0237 (5)	
C14	0.5988 (3)	0.0075 (5)	0.32986 (17)	0.0265 (6)	
C13	0.5917 (3)	0.2063 (5)	0.38184 (17)	0.0269 (6)	
C12	0.4471 (3)	0.2741 (5)	0.41913 (16)	0.0251 (5)	
C11	0.3098 (3)	0.1428 (4)	0.40203 (16)	0.0224 (5)	
C16	−0.3038 (3)	−0.1589 (5)	−0.14857 (18)	0.0255 (6)	
H1	−0.041 (3)	0.216 (6)	0.2758 (19)	0.044 (9)*	
H2	−0.307 (3)	0.526 (5)	0.1294 (16)	0.020 (6)*	
H3	−0.408 (3)	0.398 (5)	−0.0179 (16)	0.024 (6)*	
H5	−0.129 (3)	−0.164 (4)	0.0095 (14)	0.013 (6)*	
H7	0.039 (3)	−0.236 (5)	0.1538 (15)	0.021 (6)*	
H8	0.186 (3)	−0.326 (5)	0.2845 (16)	0.022 (6)*	
H15	0.466 (3)	−0.261 (5)	0.2753 (19)	0.036 (8)*	
H14	0.701 (3)	−0.044 (5)	0.3014 (16)	0.030 (7)*	
H13	0.684 (3)	0.319 (6)	0.390 (2)	0.046 (8)*	
H12	0.443 (3)	0.421 (5)	0.4568 (16)	0.028 (7)*	
H11	0.210 (3)	0.198 (5)	0.4247 (16)	0.029 (6)*	
H9A	0.045 (3)	−0.149 (5)	0.4484 (17)	0.031 (7)*	
H16A	−0.330 (3)	−0.274 (5)	−0.1053 (18)	0.031 (7)*	
H9B	−0.020 (3)	−0.371 (5)	0.3935 (16)	0.026 (6)*	
H16B	−0.191 (3)	−0.172 (4)	−0.1610 (14)	0.014 (5)*	
H9C	0.147 (3)	−0.381 (6)	0.4531 (19)	0.043 (8)*	
H16C	−0.365 (3)	−0.172 (5)	−0.2090 (18)	0.027 (7)*	

### Atomic displacement parameters (Å^2^)

**Table:**

	*U*^11^	*U*^22^	*U*^33^	*U*^12^	*U*^13^	*U*^23^
O1	0.0236 (8)	0.0249 (10)	0.0252 (9)	−0.0010 (8)	0.0002 (7)	−0.0063 (8)
O2	0.0230 (8)	0.0276 (10)	0.0198 (8)	0.0002 (7)	−0.0012 (6)	0.0021 (8)
N1	0.0171 (8)	0.0238 (11)	0.0218 (10)	−0.0012 (8)	0.0011 (7)	−0.0013 (9)
C1	0.0191 (10)	0.0217 (14)	0.0208 (12)	−0.0029 (10)	0.0046 (9)	−0.0015 (10)
C2	0.0208 (10)	0.0195 (13)	0.0306 (13)	0.0001 (10)	0.0055 (9)	−0.0011 (12)
C3	0.0144 (10)	0.0230 (13)	0.0268 (13)	0.0015 (9)	0.0023 (9)	0.0066 (11)
C4	0.0174 (10)	0.0251 (14)	0.0184 (12)	−0.0031 (9)	0.0035 (9)	0.0022 (10)
C5	0.0173 (10)	0.0210 (13)	0.0219 (12)	−0.0021 (9)	0.0033 (8)	−0.0003 (10)
C6	0.0160 (9)	0.0203 (12)	0.0209 (11)	−0.0021 (9)	0.0031 (9)	0.0001 (10)
C7	0.0155 (9)	0.0230 (13)	0.0212 (12)	−0.0017 (9)	0.0043 (8)	0.0002 (10)
C8	0.0202 (10)	0.0194 (12)	0.0215 (12)	0.0014 (9)	0.0002 (9)	−0.0014 (10)
C9	0.0233 (11)	0.0265 (14)	0.0263 (13)	−0.0046 (11)	0.0019 (10)	0.0000 (11)
C10	0.0219 (10)	0.0228 (13)	0.0149 (11)	−0.0009 (10)	−0.0015 (8)	0.0034 (10)
C15	0.0234 (11)	0.0286 (14)	0.0193 (12)	0.0033 (10)	0.0024 (9)	0.0005 (11)
C14	0.0213 (11)	0.0342 (16)	0.0242 (13)	0.0011 (10)	0.0042 (9)	0.0044 (11)
C13	0.0258 (12)	0.0322 (15)	0.0224 (13)	−0.0081 (11)	−0.0012 (10)	0.0043 (11)
C12	0.0302 (12)	0.0255 (14)	0.0192 (12)	−0.0041 (11)	−0.0014 (9)	0.0002 (11)
C11	0.0219 (11)	0.0233 (13)	0.0219 (12)	0.0021 (10)	0.0011 (9)	0.0000 (10)
C16	0.0269 (12)	0.0280 (15)	0.0217 (14)	0.0011 (11)	0.0011 (10)	−0.0009 (12)

### Geometric parameters (Å, °)

**Table:**

O1—C1	1.361 (3)	C8—H8	0.99 (3)
O1—H1	0.98 (3)	C9—H9A	0.93 (3)
O2—C4	1.374 (3)	C9—H9B	1.00 (3)
O2—C16	1.422 (3)	C9—H9C	1.02 (3)
N1—C7	1.283 (3)	C10—C15	1.393 (3)
N1—C8	1.466 (3)	C10—C11	1.393 (3)
C1—C2	1.387 (3)	C15—C14	1.390 (4)
C1—C6	1.409 (3)	C15—H15	0.96 (3)
C2—C3	1.376 (3)	C14—C13	1.379 (4)
C2—H2	0.91 (3)	C14—H14	1.00 (2)
C3—C4	1.399 (3)	C13—C12	1.389 (3)
C3—H3	0.92 (3)	C13—H13	1.01 (3)
C4—C5	1.379 (3)	C12—C11	1.383 (3)
C5—C6	1.415 (3)	C12—H12	1.01 (3)
C5—H5	0.95 (2)	C11—H11	0.95 (3)
C6—C7	1.452 (3)	C16—H16A	0.94 (3)
C7—H7	0.97 (3)	C16—H16B	0.96 (2)
C8—C10	1.519 (3)	C16—H16C	0.96 (3)
C8—C9	1.528 (3)		
			
C1—O1—H1	104.5 (17)	C8—C9—H9A	110.3 (16)
C4—O2—C16	116.9 (2)	C8—C9—H9B	111.4 (13)
C7—N1—C8	119.6 (2)	H9A—C9—H9B	111 (2)
O1—C1—C2	118.5 (2)	C8—C9—H9C	112.1 (14)
O1—C1—C6	121.4 (2)	H9A—C9—H9C	110 (2)
C2—C1—C6	120.1 (2)	H9B—C9—H9C	102 (2)
C3—C2—C1	120.0 (2)	C15—C10—C11	118.5 (2)
C3—C2—H2	119.8 (16)	C15—C10—C8	120.2 (2)
C1—C2—H2	120.2 (16)	C11—C10—C8	121.22 (19)
C2—C3—C4	120.8 (2)	C14—C15—C10	120.4 (2)
C2—C3—H3	120.4 (16)	C14—C15—H15	119.8 (16)
C4—C3—H3	118.8 (16)	C10—C15—H15	119.8 (16)
O2—C4—C5	125.1 (2)	C13—C14—C15	120.2 (2)
O2—C4—C3	114.9 (2)	C13—C14—H14	121.9 (17)
C5—C4—C3	120.0 (2)	C15—C14—H14	117.9 (17)
C4—C5—C6	119.9 (2)	C14—C13—C12	120.2 (2)
C4—C5—H5	122.7 (14)	C14—C13—H13	124.3 (17)
C6—C5—H5	117.4 (14)	C12—C13—H13	115.3 (18)
C1—C6—C5	119.1 (2)	C11—C12—C13	119.4 (2)
C1—C6—C7	121.4 (2)	C11—C12—H12	121.2 (14)
C5—C6—C7	119.4 (2)	C13—C12—H12	119.4 (14)
N1—C7—C6	121.0 (2)	C12—C11—C10	121.2 (2)
N1—C7—H7	119.8 (14)	C12—C11—H11	117.8 (17)
C6—C7—H7	119.2 (14)	C10—C11—H11	120.8 (17)
N1—C8—C10	107.1 (2)	O2—C16—H16A	109.4 (17)
N1—C8—C9	108.32 (18)	O2—C16—H16B	113.2 (14)
C10—C8—C9	113.1 (2)	H16A—C16—H16B	109 (2)
N1—C8—H8	110.1 (14)	O2—C16—H16C	106.1 (17)
C10—C8—H8	110.1 (14)	H16A—C16—H16C	112 (2)
C9—C8—H8	108.1 (14)	H16B—C16—H16C	107.6 (18)
			
O1—C1—C2—C3	179.53 (18)	C1—C6—C7—N1	2.0 (3)
C6—C1—C2—C3	−0.6 (3)	C5—C6—C7—N1	−176.18 (18)
C1—C2—C3—C4	−1.6 (3)	C7—N1—C8—C10	120.2 (2)
C16—O2—C4—C5	3.7 (3)	C7—N1—C8—C9	−117.5 (2)
C16—O2—C4—C3	−175.42 (18)	N1—C8—C10—C15	−116.8 (2)
C2—C3—C4—O2	−178.62 (18)	C9—C8—C10—C15	124.0 (2)
C2—C3—C4—C5	2.2 (3)	N1—C8—C10—C11	59.8 (3)
O2—C4—C5—C6	−179.69 (17)	C9—C8—C10—C11	−59.5 (3)
C3—C4—C5—C6	−0.6 (3)	C11—C10—C15—C14	−1.1 (3)
O1—C1—C6—C5	−177.98 (17)	C8—C10—C15—C14	175.5 (2)
C2—C1—C6—C5	2.2 (3)	C10—C15—C14—C13	0.0 (4)
O1—C1—C6—C7	3.8 (3)	C15—C14—C13—C12	1.2 (4)
C2—C1—C6—C7	−176.03 (19)	C14—C13—C12—C11	−1.3 (4)
C4—C5—C6—C1	−1.5 (3)	C13—C12—C11—C10	0.1 (4)
C4—C5—C6—C7	176.70 (19)	C15—C10—C11—C12	1.1 (3)
C8—N1—C7—C6	−179.40 (18)	C8—C10—C11—C12	−175.5 (2)

### Hydrogen-bond geometry (Å, °)

**Table:**

*D*—H···*A*	*D*—H	H···*A*	*D*···*A*	*D*—H···*A*
O1—H1···N1	0.97 (3)	1.72 (5)	2.589 (2)	151 (3)
C12—H12···Cg1^i^	1.03 (4)	2.72 (3)	3.536 (3)	137 (3)
C16—H16C···Cg1^ii^	0.98 (4)	2.71 (3)	3.563 (3)	149 (3)

Symmetry codes: (i) −*x*+1, *y*+1/2, −*z*+1; (ii) −*x*, *y*−1/2, −*z*.
